# Effects of Carvacrol on Morphogenesis and Lipase-Associated Phenotypes in Clinical Isolates of *Candida albicans*

**DOI:** 10.3390/jof12070462

**Published:** 2026-06-24

**Authors:** Iasmin Freitas Pimentel Pequeno, Larissa Alves da Silva, Luanna de Oliveira e Lima, Meryellem Bezerra Soares, Camila Mendes Soares, Raimundo Euzébio da Costa Neto, José Maria Barbosa Filho, Felipe Queiroga Sarmento Guerra, Guilherme Maranhão Chaves, Walicyranison Plínio da Silva Rocha

**Affiliations:** 1 Clinical Mycology Laboratory, Department of Pharmaceutical Sciences, Federal University of Paraiba, João Pessoa 58051-900, Paraíba, Brazil; iasminpequeno@gmail.com (I.F.P.P.); larissa.silva@academico.ufpb.br (L.A.d.S.); luanna@ltf.ufpb.br (L.d.O.e.L.); meryellem.soares@academico.ufpb.br (M.B.S.); camilamendes2314@gmail.com (C.M.S.); raimundo.neto@academico.ufpb.br (R.E.d.C.N.); fqsg@academico.ufpb.br (F.Q.S.G.); 2 Pharmaceutical Technology Laboratory, Department of Pharmaceutical Sciences, Federal University of Paraíba, João Pessoa 58051-900, Paraíba, Brazil; jbarbosa@ltf.ufpb.br; 3 Laboratory of Medical Mycology, DMIC—Department of Mycology—BC, Federal University of Pernambuco, Recife 50670-901, Pernambuco, Brazil; guilherme.chaves@ufpe.br

**Keywords:** antifungal, fungal infection, lipases, morphogenesis, terpenes

## Abstract

Background: *Candida albicans* is the main etiological agent of oral candidiasis and expresses several virulence-associated traits that contribute to tissue invasion and persistence within the host. Among these, morphogenesis and hydrolytic enzyme secretion are central to fungal pathogenicity. Carvacrol, a phenolic monoterpenoid found in essential oils from aromatic plants, has demonstrated antifungal activity against *Candida* species, although its effects on virulence phenotypes in clinical isolates remain poorly explored. Therefore, this study investigated the effects of carvacrol on morphogenesis and lipase activity in clinical isolates of *C. albicans* obtained from oral candidiasis. Methods: Thirteen clinical isolates of *C. albicans* obtained from the oral mucosa of patients with oral candidiasis and one reference strain (ATCC 90028) were evaluated in the presence and absence of carvacrol (256 μg/mL). The effects of carvacrol on germ tube formation, morphology index, hyphal length, colony filamentation in Spider medium, and lipase activity were analyzed using phenotypic assays. Results: Carvacrol reduced germ tube formation in most of the evaluated strains and decreased the overall morphology index, indicating attenuation of filamentous morphologies. In strains that maintained hyphal growth, treatment with carvacrol significantly reduced hyphal length. In addition, colonies grown in Spider medium supplemented with carvacrol exhibited predominantly smooth morphologies, with reduced filamentous formation. Lipase activity was also inhibited in all evaluated strains in the presence of the compound. Notably, variability in phenotypic response was observed among clinical isolates, particularly in strain 97, which maintained partial filamentation under treatment conditions. Conclusions: Exposure to carvacrol was associated with alterations in morphogenesis- and lipase-associated phenotypes in clinical isolates of *C. albicans* under inhibitory conditions. Because the experiments were conducted using a concentration corresponding to 2× MIC, the present findings do not allow discrimination between specific modulation of virulence-associated phenotypes and indirect effects associated with growth inhibition.

## 1. Introduction

*Candida albicans* is an opportunistic fungal pathogen that commonly colonizes the oral cavity, gastrointestinal tract, skin, and genitourinary mucosa of healthy individuals as part of the resident microbiota [1,2,3]. Under physiological conditions, the interaction between *C. albicans* and the host usually remains balanced and asymptomatic. However, alterations in host immunity, microbial composition, or local environmental conditions may favor fungal overgrowth and pathogenic transition, leading to candidiasis [2,3]. Oral candidiasis is among the most prevalent fungal infections in humans and represents an important clinical condition, particularly in immunocompromised individuals, denture wearers, patients with xerostomia, and individuals undergoing prolonged antibiotic or antineoplastic therapy [4,5,6,7]. In addition to causing discomfort and mucosal damage, oral *Candida* infections may also represent an important source for systemic dissemination in highly susceptible patients.

The pathogenicity of *C. albicans* is multifactorial and strongly associated with its remarkable phenotypic plasticity, metabolic adaptability, and ability to express multiple virulence-associated traits [2,8,9]. These virulence factors contribute to adhesion, colonization, immune evasion, tissue invasion, and persistence within the host. Among the best characterized virulence determinants are adhesion to epithelial surfaces, biofilm formation, secretion of extracellular hydrolytic enzymes, and morphogenetic transition [8,9,10,11,12]. Importantly, these pathogenic attributes are not expressed independently but rather interact in a coordinated manner during fungal colonization and infection.

Among the virulence-associated traits of *C. albicans,* morphogenesis is considered one of the most critical determinants of pathogenicity [10,11,12]. Depending on environmental stimuli, this fungus can reversibly transition between yeast cells, pseudohyphae, and true hyphae [13,14,15]. This morphological flexibility enables adaptation to different host niches and environmental conditions while contributing directly to fungal virulence. The yeast form is generally associated with dissemination and colonization, whereas filamentous forms are closely related to epithelial invasion, biofilm maturation, immune modulation, and tissue penetration [2,12]. In particular, the transition from blastoconidia to hyphal growth has been recognized as a key event during the establishment and progression of candidiasis.

The regulation of filamentation in *C. albicans* involves complex signaling pathways responsive to environmental cues such as temperature, pH, nutrient availability, and serum components [13,16]. Under appropriate inducing conditions, fungal cells initiate germ tube formation followed by hyphal elongation and filamentous colony development. Because these morphogenetic processes are strongly associated with invasive capacity and pathogenic behavior, compounds capable of interfering with filamentation have attracted considerable interest as potential strategies targeting virulence-associated traits against *Candida* infections.

In addition to morphogenetic transition, the secretion of extracellular hydrolytic enzymes also plays an important role in the virulence of *C. albicans*. These enzymes facilitate nutrient acquisition and promote host tissue invasion through the degradation of structural and membrane-associated components [8,17]. Secreted aspartyl proteinases and phospholipases have been extensively investigated due to their direct participation in tissue damage and immune evasion [18]. In contrast, comparatively less attention has been directed toward fungal lipases, despite increasing evidence supporting their contribution to pathogenicity [19,20]. Lipases are involved in lipid hydrolysis and membrane destabilization, contributing to fungal nutrition, tissue invasion, and persistence within host environments [19,20]. Some studies also suggest that lipase activity may interfere with host immune responses and contribute to the establishment of infection.

Despite advances in antifungal therapy, the management of oral candidiasis remains challenging. Polyenes and azoles are widely employed in clinical practice; however, these drugs may present important limitations, including toxicity, adverse effects, drug interactions, prolonged treatment regimens, and the emergence of resistant strains [21,22,23]. Furthermore, conventional antifungal agents primarily target fungal viability and growth, while comparatively less attention has been directed toward therapeutic approaches aimed at attenuating pathogenic phenotypes. In this context, natural compounds capable of modulating fungal pathogenicity without necessarily inducing complete growth inhibition have emerged as promising alternatives for antifungal research.

Among bioactive natural products, terpenoids derived from essential oils have attracted considerable attention due to their broad spectrum of antimicrobial properties [24,25]. Carvacrol (5-isopropyl-2-methylphenol), a phenolic monoterpenoid present in essential oils extracted from plants of the Lamiaceae family, including *Origanum vulgare* and *Origanum majorana*, is one of the most extensively investigated compounds within this class [24,26]. Previous studies have demonstrated that carvacrol exhibits antibacterial, antifungal, antioxidant, and anti-inflammatory activities [24,25,26,27,28]. In *Candida* species, this compound has been associated with alterations in membrane integrity, interference with ergosterol biosynthesis, oxidative stress induction, apoptosis-related events, and inhibition of biofilm formation [24,26,28,29,30].

Additionally, some investigations have suggested that terpenoid compounds, including carvacrol, may interfere with filamentation and morphogenetic transition in *C. albicans* [29,30]. Nevertheless, most previous studies have focused predominantly on reference strains, biofilm-related analyses, or general antifungal activity, while the phenotypic behavior of clinical isolates has been comparatively underexplored. This limitation is particularly relevant because substantial variability in virulence expression and filamentation capacity has been reported among clinical strains of *C. albicans* [31]. Therefore, evaluating the response of multiple clinical isolates may provide a broader understanding of the effects of carvacrol on virulence-associated phenotypes and its effects on filamentation-related traits.

Moreover, although previous studies have investigated the effects of natural compounds on proteinase and phospholipase secretion by *C. albicans,* evidence regarding the effects of carvacrol on fungal lipase activity remains scarce. Considering the potential contribution of lipases to fungal pathogenicity and tissue invasion, investigating this virulence-associated phenotype may contribute to a more comprehensive understanding of the biological effects of carvacrol on *C. albicans*.

Considering the increasing interest in understanding how antifungal compounds influence virulence-associated phenotypes in *Candida* spp., identifying molecules capable of altering morphogenesis and hydrolytic enzyme production may contribute to a broader understanding of fungal pathogenicity and host–pathogen interactions.

Therefore, the present study investigated the effects of carvacrol on morphogenesis and lipase activity in clinical isolates of *C. albicans* obtained from the oral mucosa of patients with oral candidiasis.

## 2. Materials and Methods

### 2.1. Carvacrol Acquisition

Carvacrol (5-isopropyl-2-methylphenol) with 98% purity was commercially obtained from Sigma-Aldrich Brasil Ltda (São Paulo, SP, Brazil).

### 2.2. Fungal Strains and Culture Conditions

Fourteen strains of *Candida albicans* were included in this study, comprising thirteen clinical isolates obtained from the oral mucosa of patients diagnosed with oral candidiasis (strains 88–100) and one reference strain (ATCC 90028). The study protocol was approved by the local Research Ethics Committee under protocol number CAAE 23611519.6.0000.5292.

All strains belonged to the yeast collection of the Clinical Mycology Laboratory, Department of Pharmaceutical Sciences, Federal University of Paraíba, Brazil. The clinical isolates used in this study correspond to strains previously characterized by our research group and reported by [30]. Although the strain codes were updated according to the current nomenclature adopted by the culture collection, they represent the same isolates described in the previous study. Species identification had been previously established using conventional phenotypic methods and further supported by molecular genotyping approaches, including ABC genotyping and microsatellite analysis. Therefore, the CHROMagar *Candida*^®^ medium (Becton Dickinson, Sparks, MD, USA) evaluation performed in the present study was used exclusively to assess culture purity and confirm phenotypic characteristics compatible with *C. albicans*.

Prior to the experiments, isolates were reactivated by two successive subcultures on Sabouraud Dextrose Agar (40 g/L dextrose, 10 g/L peptone, and 20 g/L agar) and incubated at 37 °C for 48 h. Subsequently, strain purity and colony morphology compatible with *C. albicans* were assessed on CHROMagar *Candida*^®^ medium (Becton Dickinson, Sparks, MD, USA), with green colonies being considered consistent with the species phenotype [31,32].

### 2.3. Determination of Minimum Inhibitory Concentration (MIC)

The minimum inhibitory concentration (MIC) of carvacrol against *C. albicans* strains was determined using the broth microdilution method according to the Clinical and Laboratory Standards Institute (CLSI) guidelines.

Carvacrol stock solution was prepared using Tween 80 (2%) and dimethyl sulfoxide (DMSO, 5%). Fungal suspensions were initially adjusted to approximately 1.5 × 10^6^ cells/mL and subsequently diluted according to CLSI M27-A3 recommendations to obtain a final inoculum concentration of 0.5 × 10^3^ to 2.5 × 10^3^ cells/mL in the microdilution wells. The standardized inoculum was then distributed into 96-well U-bottom microplates (Kasvi, São José dos Pinhais, PR, Brazil) containing RPMI-1640 medium supplemented with glutamine, phenol red, and MOPS buffer (3-[N-morpholino]propanesulfonic acid). The highest tested concentration of carvacrol was 1024 μg/mL. Sterility, solvent, and growth controls were included in all experiments.

Microplates were incubated at 35 °C for 24 h under ambient atmospheric conditions. MIC determination was performed according to CLSI document M27-A3 and corresponded to the lowest concentration producing a prominent reduction in visible fungal growth compared with the growth control. The predominant inhibitory concentration observed among the evaluated strains was 128 μg/mL. Additional viability assessment performed after broth microdilution assays demonstrated that detectable fungal growth was still observed at the MIC endpoint after subculture onto solid medium, indicating that the inhibitory concentration did not correspond to complete fungicidal activity.

### 2.4. Morphogenesis Assay in Liquid Medium

The effects of carvacrol on morphogenesis were evaluated under inducing conditions using YPD broth supplemented with fetal bovine serum (FBS).

Initially, *C. albicans* strains were cultured overnight (16–18 h) in YPD broth in the presence or absence of carvacrol at 256 μg/mL (2× MIC). This concentration was selected because the phenotypic assays involved prolonged exposure periods, including overnight incubation and subsequent morphogenesis induction steps. Furthermore, preliminary viability assessment performed after broth microdilution assays demonstrated that detectable fungal growth remained at the MIC endpoint following subculture onto solid medium, indicating that the MIC did not correspond to complete fungicidal activity. Therefore, 2× MIC was employed to maintain inhibitory pressure throughout the experimental protocol. Cell density was determined spectrophotometrically at 600 nm and standardized to 1 × 10^6^ cells/mL.

To induce morphogenesis, 30 μL of the standardized suspension were inoculated into tubes containing YPD broth supplemented with 20% FBS (Vitrocell Embriolife, Campinas, Brazil) and incubated at 37 °C. Samples were collected after 1 h and 3 h of incubation. At each time point, 500 μL aliquots were fixed in 10% formaldehyde solution and stored at 4 °C until microscopic analysis.

Microscopic observations were performed using an Olympus CX21 optical microscope (Olympus Corporation, Tokyo, Japan) at 400× magnification. All experiments were performed in triplicate.

### 2.5. Germ Tube Formation

For the 1 h incubation period, one hundred cells were counted per slide and the percentage of cells exhibiting germ tube emission was determined.

### 2.6. Morphology Index Determination

For the 3 h incubation period, fungal morphology was evaluated using the morphology index (MI) described by Merson-Davies and Odds [33]. One hundred cells per slide were classified according to morphological characteristics as follows: blastoconidia (MI = 1), elongated cells with diameter twice the length (MI = 2), pseudohyphae (MI = 3), and true hyphae with parallel walls (MI = 4).

The morphology index was calculated using the following equation:

MI = [(N°IM1 × 1) + (N°IM2 × 2) + (N°IM3 × 3) + (N°IM4 × 4)]/100

Values closer to 1 indicated predominantly yeast morphologies, whereas values approaching 4 indicated predominance of true hyphal forms [33,34].

### 2.7. Measurement of Hyphal Length

Hyphal length was measured in strains that exhibited morphology index values greater than 3 during the morphogenesis assay. After 3 h incubation, samples were centrifuged at 10,000 rpm for 10 min and 10 μL aliquots were transferred to microscope slides for analysis.

Hyphal measurements were performed using an optical microscope (200× magnification) coupled to the NIS-Elements D imaging software (version 6.20.00). For each strain, one hundred hyphae were measured under treated and untreated conditions. The image scale adopted in the software corresponded to 100 μm [17,35]. All experiments were performed in triplicate.

### 2.8. Morphogenesis Assay in Solid Medium

Macromorphological alterations associated with filamentation were evaluated using Spider medium.

Initially, all strains were cultured overnight in YPD broth and standardized by spectrophotometry at 600 nm. Subsequently, 10 μL aliquots of each fungal suspension were inoculated at three equidistant points onto Spider agar plates prepared either in the presence or absence of carvacrol (256 μg/mL).

Spider medium was prepared using nutrient agar (4 g), mannitol (4 g), KH_2_PO_4_ (0.8 g), agar (5.8 g), and distilled water (400 mL). Plates were incubated at 30 °C for 7 days. After incubation, colony morphology was evaluated visually with emphasis on the presence or absence of filamentous structures [17,35]. All experiments were performed in triplicate.

### 2.9. Lipase Activity Assay

Lipase activity was investigated using a solid agar-based method.

*C. albicans* cultures grown overnight in YPD broth were diluted and standardized to 2 × 10^5^ cells/mL. Ten microliters of each suspension was inoculated in triplicate onto lipase agar plates prepared either in the presence or absence of carvacrol (256 μg/mL).

Lipase agar consisted of peptone (1%), sodium chloride (5%), calcium chloride (0.01%), agar (2%), and Tween 80 (1%). Plates were incubated at 30 °C for 5 days.

After incubation, colony diameters and precipitation halos were measured using a millimeter ruler. Lipase activity was expressed as the lipase zone (LZ), calculated using the equation:

LZ = colony diameter (cm)/precipitation zone diameter (cm)

Strains presenting LZ = 1.0 were considered negative for lipase activity, whereas values lower than 1.0 indicated lipase production [36].

### 2.10. Statistical Analysis

Data were analyzed using GraphPad Prism version 3.0. Results were expressed as mean ± standard deviation. Normality was assessed using the Shapiro–Wilk test. Datasets meeting normality assumptions were analyzed using the paired Student’s *t*-test, whereas datasets that did not satisfy normality assumptions were analyzed using the Wilcoxon signed-rank test. Statistical significance was considered at *p* < 0.05 with a 95% confidence interval.

## 3. Results

### 3.1. Germ Tube Formation Assay

After 1 h incubation in YPD broth supplemented with 20% fetal bovine serum, untreated *C. albicans* strains exhibited extensive germ tube formation, with a mean ± SD of 72.27 ± 14.2%. Among the fourteen evaluated strains, twelve demonstrated germ tube formation rates above 60% under untreated conditions.

**Figure 1:**
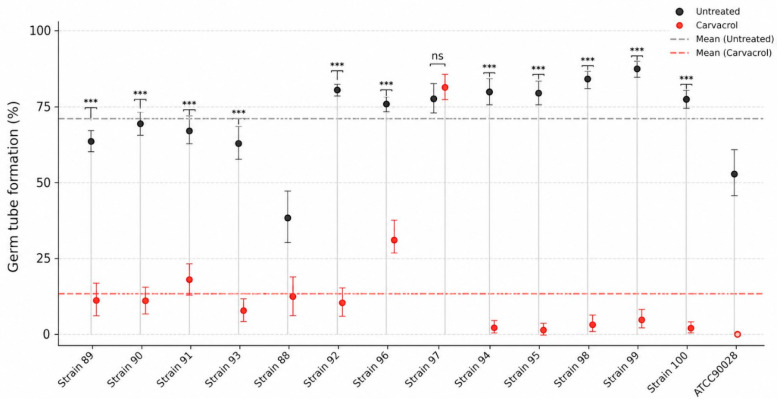
Germ tube formation by clinical isolates of *Candida albicans* following exposure to carvacrol (256 μg/mL). Untreated and treated groups are represented by black and red circles, respectively. Values correspond to mean ± SD obtained from three independent experiments. Asterisks denote statistically significant differences between groups, whereas ns indicates absence of significance (*p* < 0.05).

In contrast, exposure to carvacrol resulted in a marked reduction in germ tube formation in most clinical isolates, decreasing the overall mean emission rate (Figure 1). The most pronounced reductions were observed in strains 94, 95, 98, and 99, in which germ tube formation decreased from 82.6% to 2.3%, 82% to 1.3%, 85.6% to 2.6%, and 89% to 4%, respectively.

The reference strain ATCC 90028 also demonstrated complete inhibition of germ tube formation in the presence of carvacrol. Notably, phenotypic variability was observed among the evaluated isolates, particularly in strain 97, which maintained partial filamentation under treatment conditions. Overall, these findings indicate that exposure to carvacrol was associated with a marked reduction in the initial stages of morphogenetic transition in most evaluated clinical isolates.

### 3.2. Morphology Index Analysis

Following 3 h incubation under morphogenesis-inducing conditions, untreated strains exhibited heterogeneous cellular morphologies, including blastoconidia, pseudohyphae, germ tube-emitting cells, and true hyphae. The overall morphology index (MI) observed in untreated samples was 2.9 ± 0.6, indicating predominance of filamentous and pseudohyphal morphologies.

**Figure 2:**
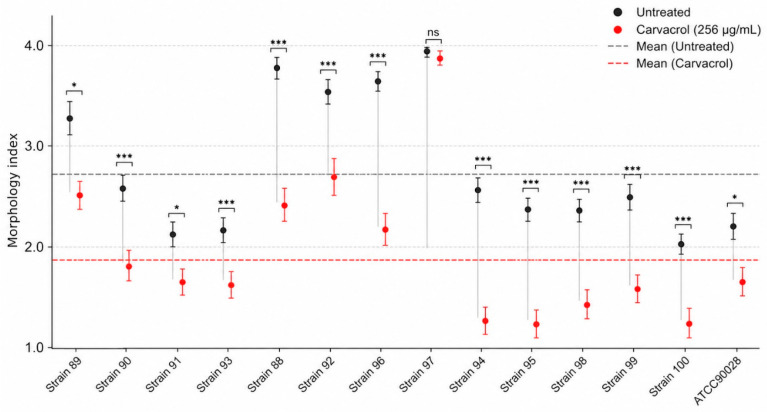
Morphology index of *Candida albicans* clinical isolates in the presence and absence of carvacrol (256 μg/mL). Black symbols correspond to untreated cells and red symbols to carvacrol-treated cells. Data are expressed as mean ± SD from three independent experiments. Significant differences are indicated by asterisks, while ns denotes non-significant variation (*p* < 0.05).

The highest MI values were observed in strains 97 (MI = 4.0 ± 0.06), 88 (MI = 3.8 ± 0.07), and 96 (MI = 3.7 ± 0.05), reflecting extensive hyphal development (Figure 2).

In the presence of carvacrol, a substantial reduction in filamentous morphologies was observed, with the overall MI decreasing to 1.9 ± 0.05. Under these conditions, most strains predominantly exhibited yeast-like and elongated cellular morphologies, associated with reduced filamentous differentiation.

**Figure 3:**
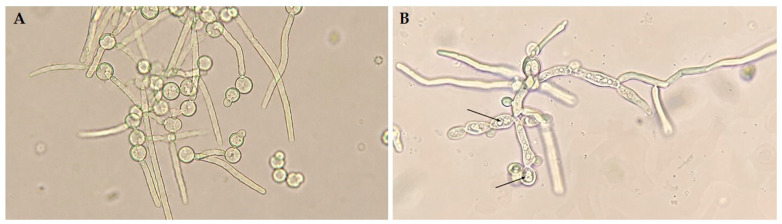
Representative microscopic morphology of *Candida albicans* cultured in YPD broth supplemented with fetal bovine serum (FBS) under filamentation-inducing conditions (400× magnification). (**A**) Untreated control exhibiting elongated filamentous structures and a relatively homogeneous cellular morphology. (**B**) Cells exposed to carvacrol (256 μg/mL), showing filamentous structures associated with morphological alterations, including enlarged cellular regions and vacuole-like intracellular structures (arrows).

Although strain 97 maintained abundant filamentous structures in the presence of carvacrol, microscopic examination revealed evident structural alterations in hyphal cells, including enlarged vacuole-like structures and cellular distension (Figure 3).

### 3.3. Hyphal Length Analysis

Hyphal length measurements were performed in strains exhibiting morphology index values greater than 3 during the morphogenesis assay. All evaluated strains demonstrated significant reductions in hyphal length following exposure to carvacrol (*p* < 0.05).

**Figure 4:**
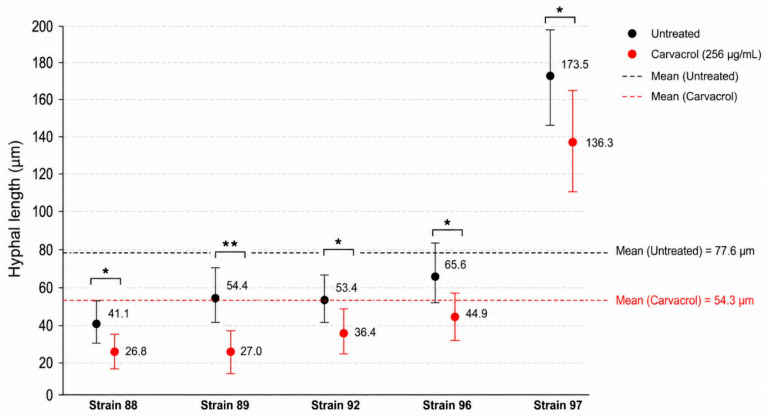
Hyphal length measurements obtained for selected clinical isolates of *Candida albicans* after treatment with carvacrol (256 μg/mL). Black and red circles indicate untreated and treated conditions, respectively. Results are presented as mean ± SD from three independent assays. Asterisks indicate statistically significant differences between experimental conditions (*p* < 0.05).

The most pronounced reduction was observed in strain 97, in which mean hyphal length decreased from 173.5 ± 44.1 μm to 136.3 ± 44.5 μm, corresponding to an average reduction of approximately 37 μm (Figure 4).

Similarly, strains 88, 89, 92, and 96 also exhibited shorter filamentous structures under treatment conditions, indicating that carvacrol was associated with reductions in both germ tube formation and hyphal elongation.

### 3.4. Morphogenesis Assay in Spider Medium

Macromorphological analysis in Spider medium demonstrated that colony filamentation was substantially reduced in the presence of carvacrol.

**Figure 5:**
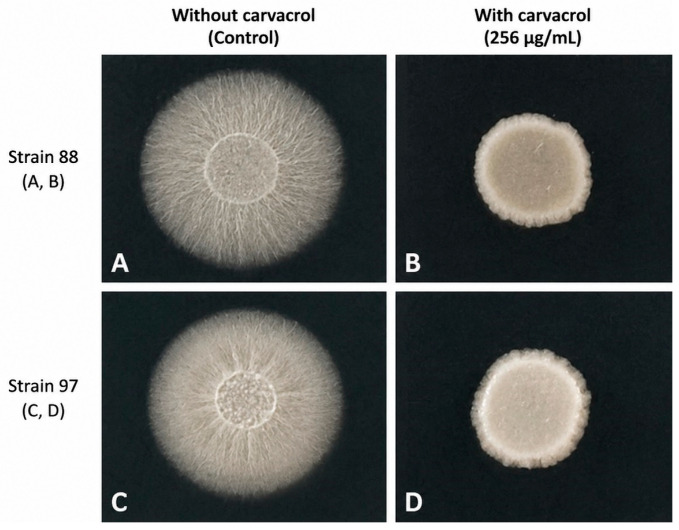
Colony morphology of *Candida albicans* isolates grown on Spider medium in the absence and presence of carvacrol (256 μg/mL). Panels (**A**,**C**) represent untreated colonies, whereas panels (**B**,**D**) represent carvacrol-treated colonies. Exposure to carvacrol was associated with reduced filamentous growth and more compact colony morphology.

Among the fourteen evaluated strains, nine exhibited filamentous colony morphology under untreated conditions. However, all filamentous colonies developed smooth macromorphological patterns when cultured in Spider medium supplemented with carvacrol (Figure 5).

The most evident phenotypic alterations were observed in strains 97 and 99, which displayed highly filamentous colonies in the absence of treatment but developed predominantly smooth colonies in the presence of carvacrol.

Overall, 64.2% of the evaluated strains demonstrated reduced filamentous colony formation following exposure to the compound.

### 3.5. Lipase Activity Assay

Lipase activity analysis revealed that eleven out of fourteen evaluated strains (78%) produced lipase under untreated conditions, as demonstrated by LZ values lower than 1.0.

**Figure 6:**
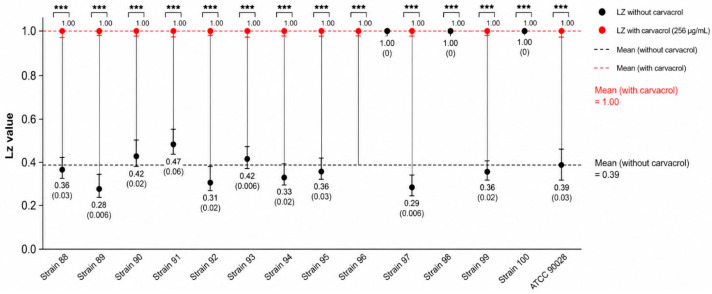
Lipase activity expressed as LZ values in clinical isolates of *Candida albicans* exposed to carvacrol (256 μg/mL). Untreated and treated groups are represented by black and red circles, respectively. Data represent mean ± SD from three independent experiments. Statistically significant differences are indicated by asterisks (*p* < 0.001).

In contrast, none of the evaluated strains exhibited detectable lipase activity in the presence of carvacrol, with all treated samples presenting LZ values equal to 1.0 (Figure 6).

These findings indicate that exposure to carvacrol was associated with absence of detectable lipase-associated phenotypes under the experimental conditions evaluated.

## 4. Discussion

The present study showed that exposure to carvacrol was associated with alterations in multiple virulence-associated phenotypes in clinical isolates of *Candida albicans*. Treatment was accompanied by reduced germ tube formation, lower morphology index values, shorter hyphal structures, suppression of filamentous colony morphology, and inhibition of lipase-associated phenotypes. Collectively, these findings indicate consistent alterations in morphogenesis- and lipase-associated phenotypes across multiple clinical isolates of *C. albicans* under the experimental conditions evaluated.

Morphogenetic transition is considered one of the most important virulence determinants in *C. albicans* pathogenicity [10,11,12]. The ability of this fungus to reversibly transition between yeast and filamentous forms contributes directly to tissue invasion, biofilm maturation, immune evasion, and persistence within host environments [2,12,16]. In the present study, carvacrol was associated with a marked reduction in germ tube formation during the early stages of filamentation. These findings are consistent with previous reports demonstrating that natural terpenoid compounds may interfere with hyphal differentiation in *Candida* species [24,29,30].

In addition to reducing germ tube emission, carvacrol also promoted substantial decreases in morphology index values, indicating attenuation of filamentous morphologies under inducing conditions. Microscopic analyses demonstrated predominance of yeast-like and elongated cellular forms following exposure to the compound, whereas untreated cells frequently exhibited pseudohyphae and true hyphae. Because filamentous growth is closely associated with invasive capacity and pathogenicity [12,16], the observed reduction in filamentous morphologies indicates that carvacrol markedly affected morphogenesis-associated phenotypes under the conditions evaluated.

The inhibitory effects of carvacrol on filamentation were further supported by hyphal length measurements and macromorphological analyses in Spider medium. Strains exposed to carvacrol exhibited shorter hyphal structures and predominantly smooth colony morphologies, whereas untreated colonies displayed filamentous borders characteristic of hyphal expansion. The consistency observed across distinct morphogenesis-related assays suggests that the observed alterations were reproducible across multiple phenotypic endpoints.

The coordinated effects observed across different phenotypic assays indicate that exposure to carvacrol was associated with alterations in traits commonly associated with *C. albicans* pathogenicity, including filamentation and lipase production. However, because these experiments were performed under inhibitory conditions, the present results do not allow definitive discrimination between specific modulation of virulence-associated pathways and indirect effects resulting from reduced cellular fitness. Consequently, the observed phenotypic alterations should be interpreted within the context of growth-inhibitory exposure conditions.

Interestingly, despite the overall reduction in filamentation, important differences in phenotypic response were observed among the evaluated clinical isolates. Strain 97 exhibited persistent germ tube formation and maintained elevated morphology index values even in the presence of carvacrol. In addition, this strain demonstrated partial preservation of filamentous colony morphology and comparatively longer hyphae under treatment conditions. The divergent phenotype observed in strain 97 may reflect the intrinsic biological heterogeneity that characterizes clinical isolates of *C. albicans*. Previous studies have demonstrated considerable strain-to-strain variability in morphogenesis, biofilm formation, virulence-associated enzyme production, stress adaptation, and susceptibility to antifungal agents. Such differences may arise from variations in genetic background, regulatory network activity, environmental adaptation, and expression of virulence-associated genes. Therefore, the persistence of partial filamentation in strain 97 is consistent with the phenotypic diversity commonly reported among clinical isolates and highlights the importance of evaluating multiple strains when investigating the biological effects of antifungal compounds.

Another relevant finding of the present study was the inhibition of lipase-associated phenotypes in all evaluated strains exposed to carvacrol. Extracellular lipases contribute to fungal nutrition, tissue invasion, membrane destabilization, and persistence within host tissues [19,20]. Although secreted proteinases and phospholipases have been extensively investigated in *Candida* pathogenicity, comparatively fewer studies have explored fungal lipases as virulence determinants [18,19,20]. Therefore, the observed suppression of lipase activity expands the understanding of the biological effects of carvacrol on *C. albicans* virulence-associated traits.

Previous studies have demonstrated that carvacrol exerts antifungal effects through multiple cellular alterations, including disruption of membrane integrity, interference with ergosterol biosynthesis, oxidative stress induction, and apoptosis-related events [24,26,28,29,30]. In addition, terpenoid compounds have been associated with alterations in fungal morphogenesis and biofilm development [29,30]. Although the present study was not designed to investigate molecular mechanisms, the observed phenotypic alterations are consistent with changes in morphogenesis- and virulence-associated phenotypes in *C. albicans.*

Some limitations of this study should be acknowledged. The phenotypic assays were performed using a concentration corresponding to 2× MIC, selected to maintain inhibitory pressure during the prolonged incubation periods required for morphogenesis induction. Although additional viability assessment performed after broth microdilution assays demonstrated that fungal growth remained detectable at the MIC endpoint following subculture onto solid medium, fungal viability was not directly assessed during the morphogenesis and lipase assays, nor was fungal growth quantitatively monitored throughout these experiments. Consequently, the relative contribution of reduced cellular fitness to the observed phenotypic alterations cannot be fully determined, and the present results do not allow definitive discrimination between specific modulation of virulence-associated phenotypes and indirect effects associated with growth inhibition. Therefore, additional studies employing subinhibitory concentrations, viability and metabolic activity measurements, CFU quantification, biomass normalization approaches, and molecular analyses are required to further characterize these effects.

Additionally, because the experiments were conducted under in vitro conditions, the biological behavior of carvacrol in host-associated environments remains uncertain. The potential cytotoxic effects of carvacrol on oral epithelial and mucosal cells were not evaluated in the present study. Previous investigations have demonstrated that the biological effects of carvacrol on mammalian cells are concentration-dependent and may vary according to the experimental model employed [37,38]. Although acceptable biocompatibility has been reported under specific conditions, particularly at lower concentrations, factors such as compound stability, retention time in the oral cavity, salivary dilution, local bioavailability, and formulation characteristics may influence its therapeutic performance in vivo. Therefore, further investigations are necessary to establish the safety profile, pharmacological feasibility, and translational applicability of carvacrol and to determine whether the concentrations associated with phenotypic modulation in vitro can be achieved and maintained under clinically relevant conditions. Accordingly, the present findings should be interpreted as evidence of phenotypic modulation under inhibitory conditions rather than direct evidence of specific virulence pathway inhibition.

Importantly, the primary objective of the present study was to characterize phenotypic alterations associated with carvacrol exposure in a collection of clinical isolates of *C. albicans* under standardized inhibitory conditions. Therefore, the experimental design was not intended to determine whether the observed effects occurred independently of growth inhibition. Addressing this question will require dedicated studies employing subinhibitory concentrations, growth-normalized approaches, and complementary in vitro and in vivo models specifically designed to evaluate virulence modulation independently of antifungal activity.

Despite these limitations, the present study showed that exposure to carvacrol was associated with alterations in multiple pathogenicity-related phenotypes in clinical isolates of *C. albicans*. The coordinated phenotypic alterations observed on filamentation-related attributes and lipase activity reinforce the potential relevance of this compound in investigations of fungal pathogenicity and highlight the importance of considering phenotypic heterogeneity among clinical isolates during investigations involving fungal pathogenicity modulation.

An additional limitation relates to the morphology index analysis. Although this approach is widely used to assess morphogenetic transitions in *C. albicans*, it represents a semiquantitative scoring system based on categorical morphological classifications. Consequently, intermediate values may not always reflect discrete biological states and should be interpreted with caution, particularly when subtle phenotypic differences are observed among clinical isolates.

An additional limitation relates to the evaluation of lipase-associated phenotypes using an agar-based precipitation assay. Although this method is widely employed for phenotypic screening of extracellular lipase activity, it provides semiquantitative information and may be influenced by factors beyond enzyme production alone. Because colony biomass, metabolic activity, and fungal viability were not normalized during the assay, the present results do not allow definitive discrimination between specific effects on lipase-associated phenotypes and indirect effects resulting from reduced cellular fitness under inhibitory conditions. Future studies employing quantitative enzymatic assays and growth-normalized approaches will be important to further characterize the effects of carvacrol on lipase expression and activity.

## 5. Conclusions

The present study demonstrated that exposure to carvacrol under inhibitory conditions was associated with consistent alterations in morphogenesis- and lipase-associated phenotypes across multiple clinical isolates of *Candida albicans*. These findings expand current knowledge regarding phenotypic alterations observed following carvacrol exposure in clinical isolates of *C. albicans.*

However, because the experiments were conducted under inhibitory conditions and fungal viability was not directly monitored during the phenotypic assays, the observed effects should be interpreted with caution. Additional studies employing subinhibitory concentrations, growth-normalized approaches, and molecular analyses are necessary to clarify the relationship between growth inhibition and the alterations in virulence-associated phenotypes observed in the present study.

## Data Availability

The datasets used and/or analysed during the current study are available from the corresponding author on reasonable request.

## Abbreviations

The following abbreviations are used in this manuscript:

ATCC	American Type Culture Collection
CAAE	Certificate of Presentation for Ethical Consideration
CLSI	Clinical and Laboratory Standards Institute
DMSO	Dimethyl sulfoxide
FBS	Fetal bovine serum
LZ	Lipase zone
MI	Morphology index
MIC	Minimum inhibitory concentration
MOPS	3-(N-morpholino) propanesulfonic acid
RPMI	Roswell Park Memorial Institute medium
SD	Standard deviation
YPD	Yeast extract peptone dextrose
